# Temporal Changes in CSF Cell Parameters After SAH: Comparison of Ventricular and Spinal Drain Samples

**DOI:** 10.1007/s12028-024-01942-2

**Published:** 2024-02-14

**Authors:** Jyri J. Virta, Jarno Satopää, Teemu Luostarinen, Jaakko Kaprio, Mika Niemelä, Miikka Korja, Rahul Raj

**Affiliations:** 1 Anesthesiology and Intensive Care, University of Helsinki and Helsinki University Hospital, Helsinki, Finland; 2 Department of Neurosurgery, Helsinki University Hospital and University of Helsinki, Helsinki, Finland; 3 Institute for Molecular Medicine Finland, University of Helsinki, Helsinki, Finland

**Keywords:** Subarachnoid hemorrhage, Cerebrospinal fluid, Inflammation, Leucocytes, Cell ratio

## Abstract

**Background:**

Forty percent of patients with aneurysmatic subarachnoid hemorrhage (aSAH) develop acute hydrocephalus requiring treatment with cerebrospinal fluid (CSF) drainage. CSF cell parameters are used in the diagnosis of nosocomial infections but also reflect sterile inflammation after aSAH. We aimed to study the temporal changes in CSF parameters and compare external ventricular drain (EVD)–derived and lumbar spinal drain–derived samples.

**Methods:**

We retrospectively identified consecutive patients with aSAH treated at our neurointensive care unit between January 2014 and May 2019. We mapped the temporal changes in CSF leucocyte count, erythrocyte count, cell ratio, and cell index during the first 19 days after aSAH separately for EVD-derived and spinal drain–derived samples. We compared the sample sources using a linear mixed model, controlling for repeated sampling.

**Results:**

We included 1360 CSF samples from 197 patients in the analyses. In EVD-derived samples, the CSF leucocyte count peaked at days 4–5 after aSAH, reaching a median of 225 × 10^6^ (interquartile range [IQR] 64–618 × 10^6^). The cell ratio and index peaked at 8–9 days (0.90% [IQR 0.35–1.98%] and 2.71 [IQR 1.25–6.73], respectively). In spinal drain–derived samples, the leucocyte count peaked at days 6–7, reaching a median of 238 × 10^6^ (IQR 60–396 × 10^6^). The cell ratio and index peaked at 14–15 days (4.12% [IQR 0.63–10.61%]) and 12–13 days after aSAH (8.84 [IQR 3.73–18.84]), respectively. Compared to EVD-derived samples, the leucocyte count was significantly higher in spinal drain–derived samples at days 6–17, and the cell ratio as well as the cell index was significantly higher in spinal drain–derived samples compared to EVD samples at days 10–15.

**Conclusions:**

CSF cell parameters undergo dynamic temporal changes after aSAH. CSF samples from different CSF compartments are not comparable.

**Supplementary Information:**

The online version contains supplementary material available at 10.1007/s12028-024-01942-2.

## Introduction

Aneurysmal subarachnoid hemorrhage (aSAH) is a devastating form of stroke with an estimated case fatality rate of 40% [1] and significant long-term disability in survivors. The pathophysiology of aSAH and delayed cerebral ischemia, one of its major complications, is complex and multifactorial, including excitotoxicity, microthrombi, blood–brain barrier disruption, and neuroinflammation [2]. Forty percent of patients with aSAH have acute hydrocephalus at admission [3], and this can be treated in the acute to subacute phase with an external ventricular drain (EVD) and/or lumbar spinal drainage. Repeated cerebrospinal fluid (CSF) samples from either compartment provide information about the changes in CSF cell parameters as aSAH develops. Knowledge about this development may help in assessing inflammatory and infectious processes during intensive care.

The CSF leucocyte/erythrocyte ratio (cell ratio) and the CSF leucocyte/erytrocyte to peripheral blood leucocyte/erytrocyte ratio (cell index) are used routinely in the diagnostics of nocosomial CSF infections after aSAH [4]. However, the cell ratio and index can also be seen to reflect the brain’s neuroinflammatory response after SAH. Previous studies suggest that the CSF erythrocyte count peaks at 2–3 days, the CSF leucocyte count peaks at 5–8 days, and the CSF cell ratio and cell index peak at 12–15 days after aSAH [5–8]. However, the studies have been limited by their small sample sizes. Additionally, they either have included only EVD-derived samples [5, 7, 8] or have not separated EVD and spinal drain samples [6]. In the current study, we aimed to describe the temporal changes in CSF cell parameters during the first 19 days after aSAH separately for EVD-derived and spinal drain–derived samples. We also compared the parameters between the different CSF compartments and hypothesized that the findings are not equal.

## Methods

### Study Setting and Cohort

We conducted a retrospective single-center study of consecutive patients with aSAH admitted to the neurointensive care unit (neuro-ICU) of Helsinki University Hospital between January 2014 and May 2019. We screened all admitted patients with a diagnosis of nontraumatic SAH and included only those with verified aSAH. We have described the cohort in more detail [9]. We excluded patients who were admitted > 24 h after ictus and patients who were transferred to or from another unit during CSF drainage.

Our standard protocol includes primary treatment with an EVD, and a spinal drain is rarely used as a first-line option. The EVD can later be converted to a spinal drain if EVD weaning fails repeatedly. A failed EVD weaning is considered when the patient’s clinical condition deteriorates and/or intracranial pressure rises repeatedly to > 20–25 mm Hg following EVD closure, which is commonly tried more than once and for a few days. Also, patients with no or mild neurological deficits can be occasionally treated with a spinal drain if they develop late-onset (> 1 week) symptomatic and nonobstructive post-SAH hydrocephalus. More recently, a direct shunt operation is sometimes considered for patients with failed EVD weaning or late-onset hydrocephalus.

CSF samples are routinely taken three times per week and on clinical suspicion of nosocomial central nervous system (CNS) infection. The samples include cell counts and a proportion of neutrophils of leucocytes but do not include CSF glucose or protein concentrations.

The local institutional research committee approved the study and waived the need for patient consent (HUS/466/2019 §106). The article was prepared according to Strengthening the Reporting of Observational Studies in Epidemiology guidelines.

### Data Collection

We retrospectively extracted the following data from health records and imaging studies: age at admission, sex, World Federation of Neurological Surgeons (WFNS) grade on admission [10], the presence of a thick and diffuse bleeding pattern (clot thickness ≥ 4 mm in ≥ 3 cisterns), any intraventricular hemorrhage (IVH) regardless of volume or number of ventricles affected, aneurysm treatment modality (endovascular or surgical), diagnosis of delayed cerebral ischemia based on clinical and/or radiological findings [9], and 12-month Glasgow Outcome Scale [11]. We identified patients who received an EVD or spinal drain and collected data on insertion and removal dates and CSF culture results and dates.

### CSF Samples

Helsinki University Hospital administers HUS Data Lake, which is a health care data repository that includes patient-level data from multiple electronic health record systems, including laboratory values. We queried the database to obtain the following laboratory values taken within 19 days of admission: CSF leucocyte count (in 10^6^/L), CSF erythrocyte count (10^6^/L), proportion of neutrophils in CSF (available only if leucocyte count was > 19 × 10^6^/L), blood leucocyte count (10^9^/L), and blood erythrocyte count (10^12^/L).

We included only samples that could be reliably identified as either EVD derived or spinal drain derived, that is, we excluded samples taken on a day when the EVD was converted to a spinal drain because we could not verify which drain the samples were taken from. Additionally, we excluded samples taken within 3 days before or any time after a positive CSF culture result to exclude samples possibly representing nosocomial EVD-associated infection. If a patient had two or more samples available for the same day, we used the mean of these values.

We calculated the CSF cell ratio (leucocyte count/erythrocyte count) and cell index [(CSF leucocyte count/CSF erythrocyte count)/(blood leucocyte count/blood erythrocyte count)]. Because low CSF erythrocyte counts can result in an unproportionally high cell ratio and cell index, we excluded samples that had a CSF erythrocyte count lower than the fifth percentile from analyses on the cell ratio and cell index. A cell index > 1 indicates a higher leucocyte count in CSF than in peripheral blood.

### Statistical Analyses

We report means ± standard deviations (SDs) for normally distributed variables and medians with interquartile ranges (IQRs) for variables with a skewed distribution. For categorical variables, we report numbers of observations and proportions.

For CSF analyses, the samples were grouped into 2-day periods (e.g., samples taken on days 0–1, 2–3, etc., after aSAH). The CSF erythrocyte count, leucocyte count, cell ratio, and cell index were strongly skewed to the right. Therefore, we report crude median values with IQRs for these values within the 19 days after admission. In contrast, the proportion of neutrophils had a normal distribution, and we report means with SDs for the proportion.

We used a linear mixed model, accounting for repeated sampling, to assess the temporal development of the CSF parameters and to compare EVD-derived and spinal drain–derived samples. Because the number of EVD samples was low after 17 days following admission and the number of spinal drain samples was low during the first 5 days after admission, we included only days 6–17 in the model. We assumed that the temporal development of CSF parameters was not linear and, hence, included a quadratic term (i.e., time squared) in the model. Because of the strongly skewed distributions, we log-transformed the CSF values for modeling, except for the proportion of neutrophils. We assessed the validity of model assumptions with analysis of residuals.

To test for possible differences between EVD-derived and spinal drain–derived samples, we included the CSF compartment (EVD derived or spinal drain derived) as well as its interaction terms with time and time squared in the model. Additionally, based on biological plausibility as a contributor to the CSF parameters, we included age (as a continuous variable), presence of a thick and diffuse bleeding pattern, presence of IVH, treatment modality (surgical or endovascular), and SAH severity (WFNS grades I–III or IV–V) as fixed effect covariates.

As an additional analysis, we compared individual patients’ last EVD samples with their first spinal drain samples. These analyses were limited to patients with a maximum of 5 days between the samples. The values were compared with the Wilcoxon signed-rank test.

Because of the small number of missing values, we excluded samples with missing values from the analyses. We considered two-tailed *p* < 0.05 statistically significant. We used Stata 17.0 (StataCorp, College Station, TX) for the analyses.

## Results

### Patient and CSF Sample Characteristics

**Fig. 1:**
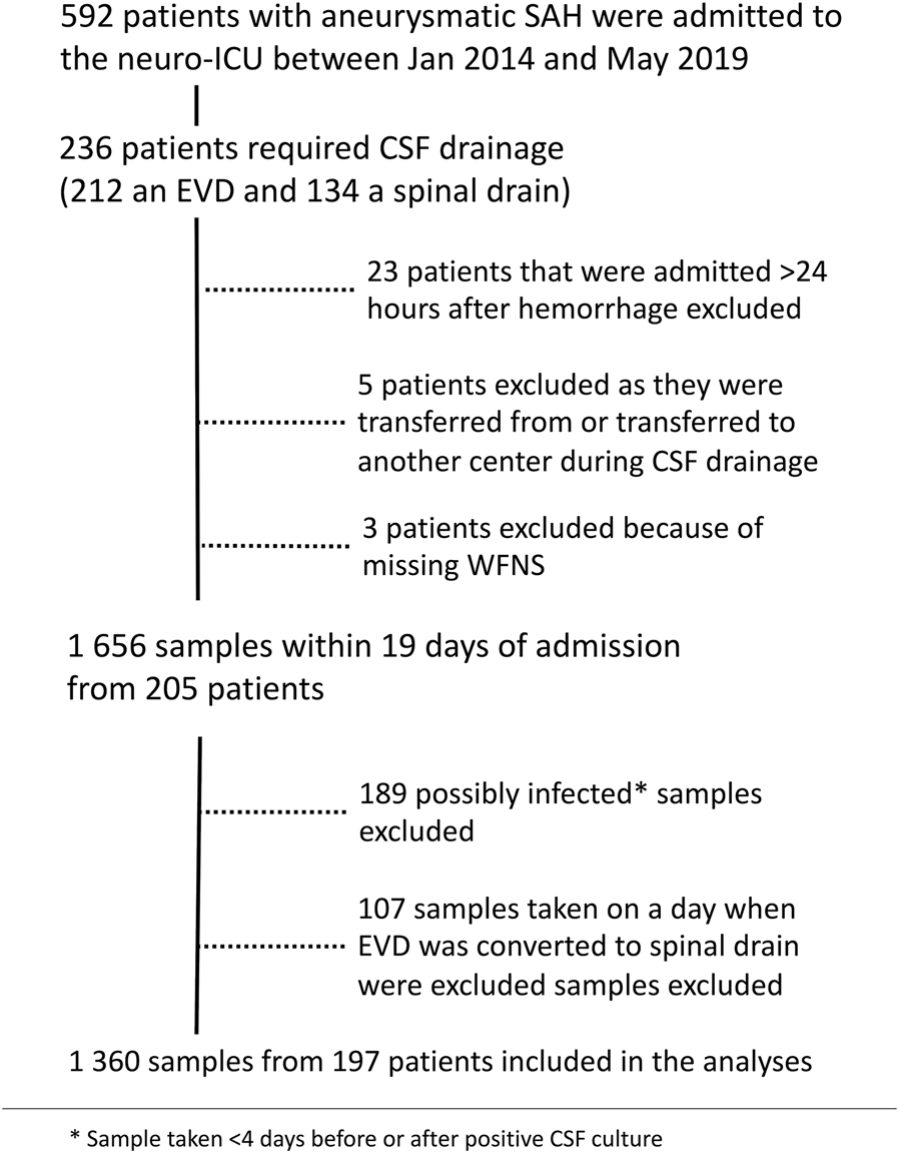
Flow chart of patients’ selection and exclusion criteria. CSF cerebrospinal fluid, EVD external ventricular drain, neuro-ICU neurointensive care unit, SAH subarachnoid hemorrhage, WFNS World Federation of Neurological Surgeons grade

Between January 2014 and May 2019, there were 592 patients with aSAH admitted to our neuro-ICU, and 236 (40%) of them required CSF drainage. Twenty-three patients were excluded because they were admitted > 24 h after ictus, three were excluded because of nonassessable WFNS grades at admission, and five were excluded because they were transferred from or to another center during CSF drainage. The study flowchart is shown in Fig. 1. Compared to the included patients, the excluded patients did not differ in age, sex, or aSAH severity.

Finally, 1656 CSF samples taken within 19 days of admission from 205 patients were considered for analysis. From these, 189 possibly infected samples were excluded, and 107 samples taken on a day when a patient’s EVD was converted to a spinal drain were excluded, leaving 1360 CSF samples from 197 patients for the analyses (Fig. 1).

Characteristics of the included patients and CSF samples are shown in Table 1. Briefly, the mean age was 60 ± 11 years, the majority (63%) were women, 55% had poor-grade aSAH (WFNS grades IV–V), and 65% received endovascular aneurysm treatment. The median time from admission to EVD placement was 0 days (IQR 0–1), and the median time from admission to spinal drain placement was 11 days (IQR 7–15). The daily number of patients with CSF drainage is shown in Supplemental Fig. 1.

**Table 1:** Patient and cerebrospinal fluid sample characteristics

	Value
*Patient characteristics*	
Number of patients, *N*	197
Age in years, mean ± SD	60 ± 11
Female patients, *n* (%)	125 (63)
WFNS grade, *n* (%)	
I	42 (21)
II	38 (19)
III	9 (5)
IV	44 (22)
V	64 (32)
Modified Fisher grade, *n* (%)	
Grade 1	13 (7)
Grade 2	35 (18)
Grade 3	27 (14)
Grade 4	122 (62)
Aneurysm treatment modality, *n* (%)	
Endovascular	128 (65)
Surgical	69 (35)
Delayed cerebral ischemia, *n* (%)	95 (48)
Glasgow Outcome Scale at 12 months^a^, *n* (%)	
Dead	35 (18)
Neurodegenerative state	1 (1)
Severe disability	44 (23)
Moderate disability	52 (27)
Good recovery	58 (31)
*CSF sample characteristics*	
Number of CSF samples, *N*	1360
EVD samples, *n* (%)	1064 (78)
Spinal drain samples, *n* (%)	296 (22)
Number of samples per patient, mean ± SD	6.9 ± 3.8
Days from SAH to CSF sample, mean ± SD	
All samples	7.9 ± 5.0
EVD samples^b^	6.5 ± 4.3
Spinal drain samples	12.9 ± 4.0

CSF, cerebrospinal fluid, EVD, external ventricular drain, SAH, subarachnoid hemorrhage, WFNS, World Federation of Neurosurgical Societies

^a^Missing for seven patients

^b^EVD samples taken significantly earlier (*p* < 0.01)

Of the 1360 CSF samples, 78% were EVD derived, and 22% spinal drain derived. The mean number of samples per patient was 6.9 ± 3.8, and the mean time from admission to CSF sample date was 7.9 ± 5.0 days. EVD-derived samples were taken significantly earlier (6.5 ± 4.3 vs. 12.9 ± 4.0 days after admission; *p* < 0.01).

### Temporal Course of CSF Parameters and Differences Between EVD-Derived and Spinal Drain–Derived Samples

**Fig. 2:**
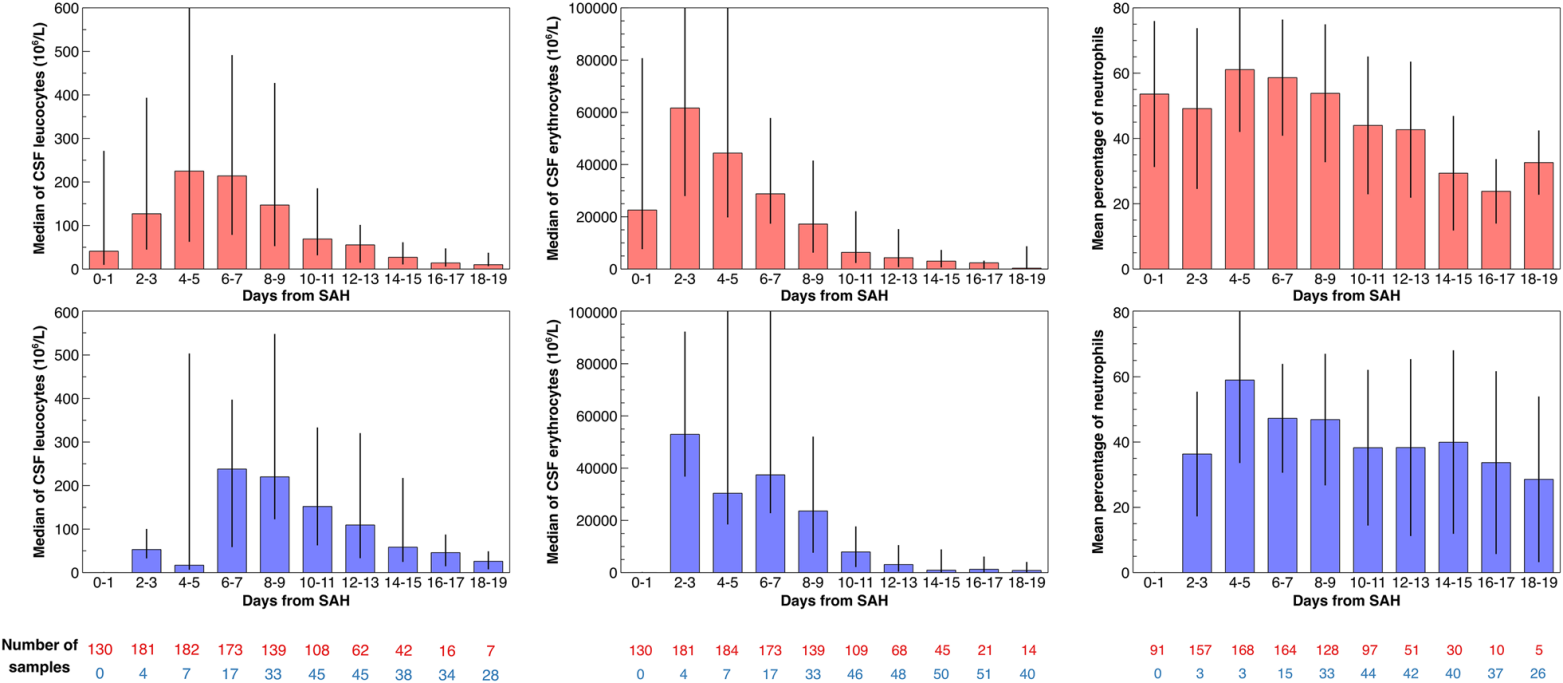
The CSF cell counts for leucocytes (left) and erythrocytes (middle) and the proportion of neutrophils (right) are shown. The upper graphs (red) show findings for external ventricular drain–derived samples, and the lower graphs (blue) show findings for spinal drain–derived samples. For leucocytes and erythrocytes, medians with interquartile ranges are shown. For the proportion of neutrophils, means with standard deviations are shown. The leucocyte count was significantly higher in spinal drain–derived samples on days 6–17, and the erythrocyte count was significantly higher in spinal drain–derived samples on days 6–11 after hemorrhage. CSF cerebrospinal fluid, SAH subarachnoid hemorrhage (Color figure online)

The CSF findings and the results of the mixed model comparing EVD-derived and spinal drain–derived samples for the CSF leucocyte count, erythrocyte count, and proportion of neutrophils are shown in Fig. 2. In EVD-derived samples, the leucocyte count reached a median of 225 (IQR 64–618) at days 4–5 after admission, followed by a gradual decline. Likewise, in spinal drain–derived samples, the leucocyte count had a maximum of 238 (IQR 60–396) at 6–7 days after admission. During days 6–17 after admission (the range included in the model comparing EVD-derived and spinal drain–derived samples), the leucocyte count was significantly higher in spinal drain–derived samples at 6–17 days after admission.

The erythrocyte count peaked at days 2–3 after admission in both sample sources, with a median count of 61,667 (IQR 28,160–167,360) in EVD-derived samples and 52,960 (IQR 37,000–91,894) in spinal drain–derived samples. The following gradual decline seemed faster in spinal drain–derived samples on visual inspection. The erythrocyte count was significantly higher in spinal drain–derived samples at 6–11 days after admission.

The proportion of CSF neutrophils was similar in EVD-derived and spinal drain–derived samples and reached its maximum in both at 4–5 days after admission (mean 61.1 ± 18.9% in EVD-derived samples and 59.0 ± 25.2% in spinal drain–derived samples) followed by a gradual decline. The values did not differ significantly at any point.

**Fig. 3:**
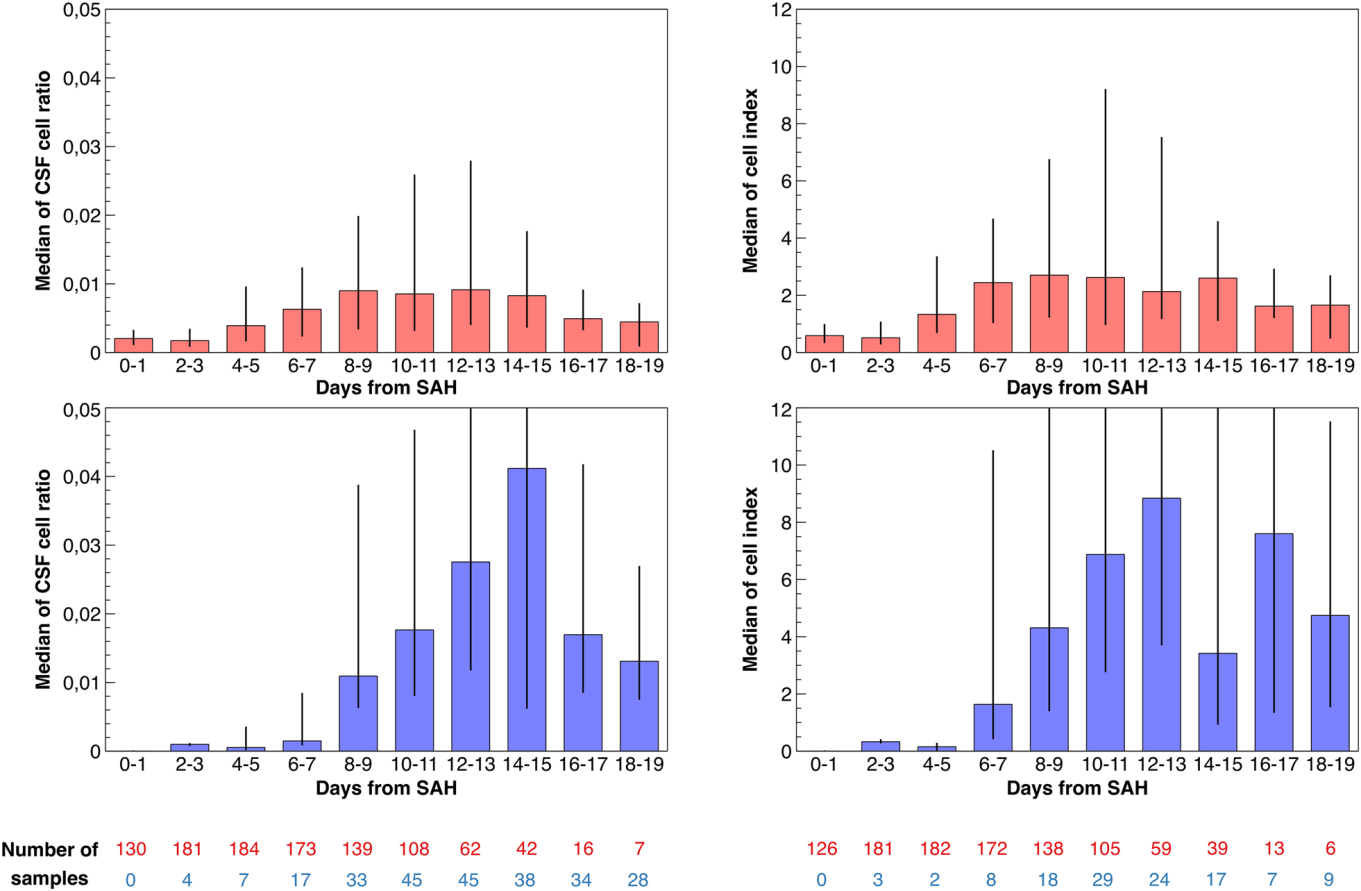
The cell ratio (left) and cell index (right) are shown. The upper graphs (red) show findings for external ventricular drain–derived samples, and the lower graphs (blue) show findings for spinal drain–derived samples. The cell ratio and cell index were significantly higher in spinal drain–derived samples on days 10–15 after hemorrhage. CSF cerebrospinal fluid, SAH subarachnoid hemorrhage (Color figure online)

The CSF findings and the results of the mixed model comparing EVD-derived and spinal drain–derived samples for the CSF cell ratio and cell index are shown in Fig. 3. In EVD-derived samples, the CSF cell ratio reached 0.90% (IQR 0.35–1.98%) at days 8–9 and remained somewhat similar until days 14–15, followed by a gradual decline. In contrast, in spinal drain–derived samples, the cell ratio increased until days 14–15, reaching a median of 4.12% (IQR 0.63–10.61%), followed by faster decline. In the model comparing the sample sources, the cell ratio was significantly higher in spinal drain–derived samples at days 10–15 after admission.

Similar to the cell ratio, the cell index reached a median of 2.71 (IQR 1.25–6.73) at days 8–9 in EVD-derived samples and remained stable until days 14–15. Again, spinal drain–derived samples reached their maximum later at days 12–13 (median 8.84 [IQR 3.73–18.84]). The cell index was significantly higher in spinal drain–derived samples at days 10–15.

Because it could be argued that patients who require both an EVD early after their SAH and a spinal drain after unsuccessful weaning do not represent all patients with aSAH, we performed a sensitivity analysis including only the 93 patients treated with both an EVD and a spinal drain. The results are shown in Supplemental Fig. 2 and did not differ from the results of the whole cohort. A comparison between patients who were treated with only an EVD, only a spinal drain, or both an EVD and a spinal drain is shown in Supplemental Table 1. No significant differences were seen in clinical or radiological characteristics between patients treated with only an EVD and patients who required both an EVD and a spinal drain.

### Comparison Between Individual Patients’ EVD and Spinal Drain Samples

Fifty-seven patients with both kinds of samples were included in the analyses. The last EVD sample was taken 8.7 ± 3.8 days after admission, and the first spinal drain sample was taken 12.0 ± 3.7 days after admission (*p* < 0.001). The CSF erythrocyte count did not significantly differ between the EVD-derived and spinal drain–derived samples (17,915 [IQR 3625–31,800] and 18,050 [IQR 5900–37,600], respectively; *p* = 0.74). In contrast, the CSF leucocyte count was significantly lower in the EVD-derived samples than in spinal drain–derived samples (86 [IQR 40–240] and 240 [IQR 84–502], respectively; *p* < 0.001). The cell ratio was also lower in EVD-derived samples than in spinal drain–derived samples (0.65% [IQR 0.28–2.33%] and 1.37% [0.59–4.65%], respectively; *p* = 0.01). Likewise, the cell index was lower in EVD-derived samples than in spinal drain–derived samples (2.7 [IQR 1.0–7.4] and 4.2 [IQR 2.4–20.2], respectively; *p* = 0.01).

## Discussion

### Main Findings

In EVD-derived CSF samples, we identified a modest increase in the erythrocyte count, reaching its maximum at days 2–3 after hemorrhage. For CSF leucocytes, a more prominent increase early after hemorrhage was seen, and the leucocyte count peaked at 4–7 days after ictus. The CSF cell ratio and index reached their maximums even later and started to decrease only after 2 weeks.

Spinal drain–derived samples differed significantly from the EVD-derived samples. The leucocyte count was significantly higher in the spinal drain-derived samples after the first week and was followed by a slower decrease afterward. Additionally, the CSF cell ratio and index were significantly higher in spinal drain–derived samples after the first 10 days and seemed to peak only at 2 weeks after hemorrhage.

### Comparison to Previous Literature

Our results are in line with previous studies that included only EVD-derived samples or EVD-derived and spinal drain–derived samples analyzed together. The erythrocyte count has been found to peak 2–3 days after SAH in most [5, 7] but not all [6, 8] studies. This early increase can be attributed to redistribution of blood in CSF spaces. Likewise, CSF leucocyte counts have increased until days 5–8 after aSAH in previous studies [6, 8], and the cell ratio [6] as well as the cell index [8] have been found to peak 12–15 days after aSAH. The only previous study examining the proportion of granulocytes found that it reaches its maximum 5–8 days after aSAH [8], which is in line with our finding regarding the proportion of neutrophils.

To our knowledge, this is the first study to systematically compare the differences between EVD-derived and spinal drain–derived CSF samples following aSAH at various time points after ictus. However, some small studies have compared CSF findings between CSF compartments in various CNS disorders and have found a craniocaudal gradient with a higher leucocyte concentration in the spinal CSF compartment [12–14].

Our results show that CSF cell counts undergo significant dynamic changes following aSAH. The sustained increases in the leukocyte count, cell ratio, and cell index indicate that CSF leucocytes are not merely peripheral cells that enter the CSF at aneurysm rupture and instead reflect a neuroinflammatory process following the initial aneurysm rupture. Additionally, our results add to the evidence that EVD-derived and spinal drain–derived CSF samples are not comparable and that their development after aSAH differs significantly. CSF is constantly produced in the ventricles and is thought to flow toward the spinal CSF spaces. Therefore, CSF circulation is slower in the lumbar CSF spaces, possibly causing the observed craniocaudal gradient seen in cell counts and ratios.

Our findings have indications for the analysis of CSF samples in patients with suspected nosocomial CNS infection associated with CSF drainage. For example, the widely used Centers for Disease Control and Prevention National Healthcare Safety Network (CDC/NHSN) criteria for nosocomial ventriculitis/meningitis do not require a positive CSF culture result, and a febrile patient with an increased CSF leucocyte count fulfills the diagnostic criteria [15]. Even when excluding patients with a positive CSF culture result, we found that the leucocyte count, cell ratio, and cell index peaked at 7–14 days after ictus. Because fever is common following SAH, especially within a similar time frame [16], it is likely that the CDC criteria overestimate the true infection rate, and a large proportion of patients meeting the criteria merely reflects “sterile” inflammation after hemorrhage. Knowledge of the natural temporal change of CSF cell parameters seems crucial to avoid unnecessary and prolonged antimicrobial treatments.

### Strengths and Limitations

Our study has several strengths. We were able to analyze a large consecutive series of patients with aSAH treated at a specialized neuro-ICU. We had extensive, although retrospectively compiled from hospital records, data on the patients’ characteristics at admission and during hospitalization, which allowed us to control for several factors plausibly affecting initial CSF cell counts. We did not have data on CSF protein levels, even though previous studies have suggested the CSF protein level to be associated with outcome [7] or shunt dependency [5] after aSAH.

The protocol of our neuro-ICU includes routine CSF samples three times a week, which reduces the risk of sampling bias. Still, some samples were taken because of suspected infection. We could not exclude samples possibly representing nosocomial infection based on clinical markers such as fever or use of antibiotics for suspected CNS infections, nor did we have data on antibiotic use for other infections, and this could have affected the number of positive CSF culture results. We did, however, exclude samples taken fewer than 3 days before a positive CSF culture result to minimize the risk of including infected samples.

Most of the patients in our cohort required CSF drainage because of acute hydrocephalus and, therefore, our patients represent the more severe end of the aSAH clinical spectrum, which is reflected by their relatively high WFNS grades. Hence, our results may not be generalizable to all patients with aSAH. As a results of variable clot resolution, individual erythrocyte and leucocyte counts varied greatly from day to day, which increased the confidence intervals of our estimates. At our institution, patients with an IVH obstructing the third or fourth ventricle are treated with intrathecal alteplase, but we could not assess whether this affected the CSF parameters. Additionally, we did not control for the CSF volume that was drained, but in a previous study, this did not significantly affect CSF cell counts [8].

We did not have CSF samples taken simultaneously from different CSF compartments. Only a small minority of our patients were treated with only a spinal drain. It is likely that patients who were initially treated with an EVD and subsequently required a spinal drain because of weaning failure do not represent all patients with aSAH. However, these patients did not seem to differ in clinical or radiological SAH severity or functional outcomes from patients treated with only an EVD.

Considering the recently published EARLYDRAIN trial showing that prophylactic early lumbar drainage decreases the rate of unfavorable outcome after aSAH [17], it is possible that the use of early lumbar drainage increases in the future. Hopefully, we will also see studies comparing CSF samples taken simultaneously from different compartments, which will enable future studies comparing CSF samples taken simultaneously from different compartments.

## Conclusions

Overall, our results add to the evidence that CSF cell counts, the cell ratio, and the cell index are not constant following aSAH, but instead they show dynamic temporal changes reflecting an inflammatory response to the hemorrhage. Given that we excluded patients with EVD-related CSF infections, our findings may have implications for diagnosis of nosocomial infections during CSF drainage. Additionally, our results indicate that samples from different CSF compartments are not comparable.

### Supplementary Information

Below is the link to the electronic supplementary material.

Supplementary file1 (PDF 116 kb)

Supplementary file2 (PDF 177 kb)

Supplementary file3 (PDF 146 kb)

Supplementary file4 (DOCX 33 kb)
