# Supplementary material for: Temporal Changes in CSF Cell Parameters After SAH: Comparison of Ventricular and Spinal Drain Samples
Source: Neurocrit Care. 2024 Feb 14;41(1):194–201. doi: 10.1007/s12028-024-01942-2 (PMC11335821; doi:10.1007/s12028-024-01942-2)

Supplemental Figure 1

The number of patients with with either an external ventricular or spinal drain following subarachnoid hemorrhage.

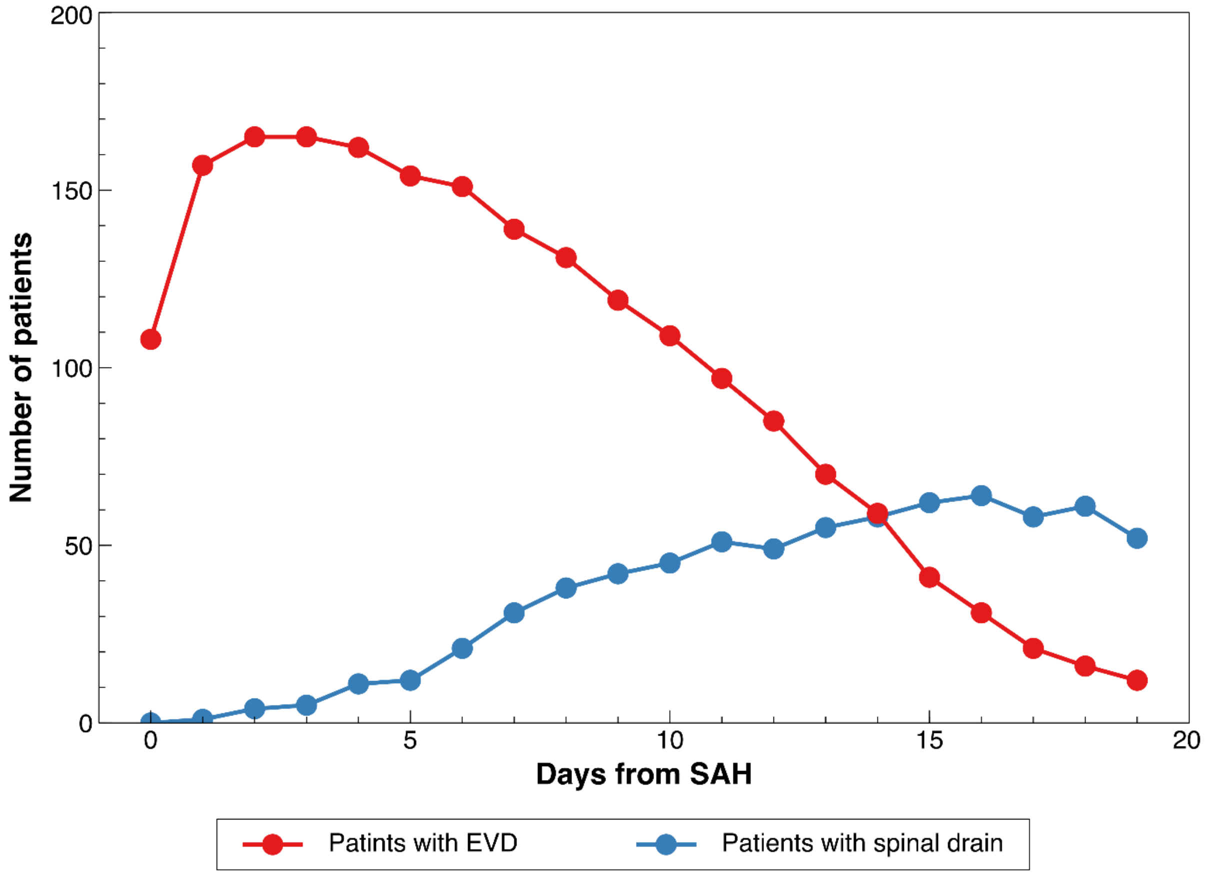

Supplement: Supplementary file 1 — Supplementary file1 (PDF 116 kb) [file 12028_2024_1942_MOESM1_ESM.pdf]
