# Supplementary material for: Temporal Changes in CSF Cell Parameters After SAH: Comparison of Ventricular and Spinal Drain Samples
Source: Neurocrit Care. 2024 Feb 14;41(1):194–201. doi: 10.1007/s12028-024-01942-2 (PMC11335821; doi:10.1007/s12028-024-01942-2)

Supplemental Figure

Temporal changes of CSF cell parameters following SAH in 93 patients requiring both and external ventricular drain and spinal drain. The red bars indicate external ventricular drain samples and the blue bars spinal drain samples. Median values with interquartile ranges are shown.

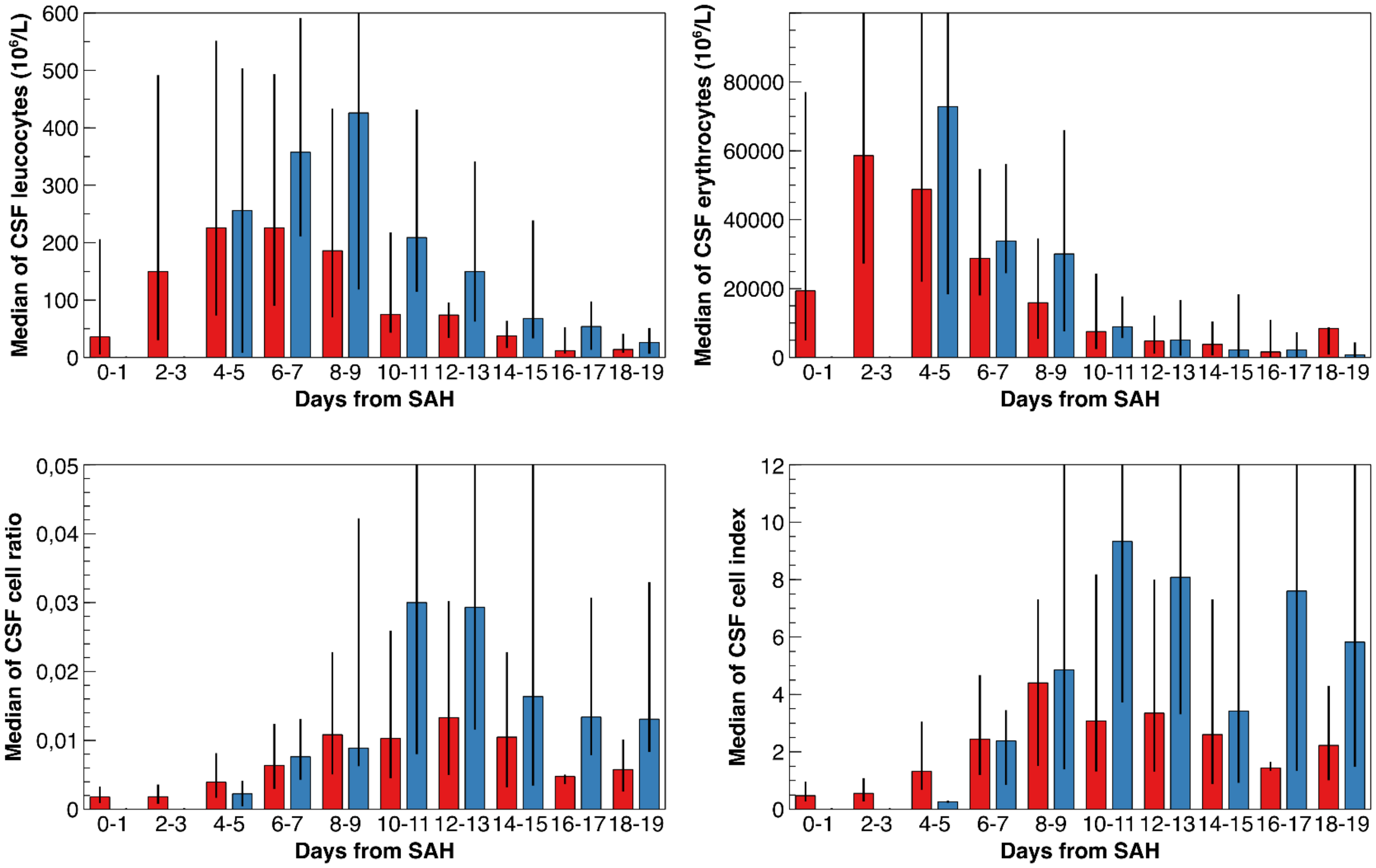

Supplement: Supplementary file 2 — Supplementary file2 (PDF 177 kb) [file 12028_2024_1942_MOESM2_ESM.pdf]
