# Supplementary material for: Temporal Changes in CSF Cell Parameters After SAH: Comparison of Ventricular and Spinal Drain Samples
Source: Neurocrit Care. 2024 Feb 14;41(1):194–201. doi: 10.1007/s12028-024-01942-2 (PMC11335821; doi:10.1007/s12028-024-01942-2)
Supplement: Supplementary file 3 — Supplementary file3 (PDF 146 kb) [file 12028_2024_1942_MOESM3_ESM.pdf]

Supplemental Table

Details on patients who were treated only with an external ventricular drain (EVD), both and EVD and a spinal drain, and only with a spinal drain. P values for a comparison between the first two groups are shown. The used statistical tests included independent samples t test (age), Chi-squared test (sex, treatment modality and delayed cerebral ischemia), and ordinal logistic regression (WFNS, modified Fisher grade and Glasgow Outcome Scale)

		**Only EVD (n=85)**	**EVD and spinal drain (n=93)**	**Only spinal drain (n=19)**	**p-value**
Age in years, mean ± SD		58 ± 12	61 ± 10	68 ± 12	0.09
Female patients, n (%)		49 (58 %)	62 (67 %)	14 (74 %)	0.22
WFNS grade, n (%)					0.72
	I	18 (21 %)	16 (17 %)	8 (42 %)	
	II	12 (14 %)	20 (21 %)	6 (32 %)	
	III	4 (5 %)	3 (3 %)	2 (11 %)	
	IV	19 (22 %)	24 (26 %)	1 (5 %)	
	V	32 (38 %)	30 (32 %)	2 (11 %)	
Modified Fisher grade, n (%)					0.19
	Grade 1	6 (7 %)	5 (5 %)	2 (10 %)	
	Grade 2	18 (21 %)	13 (14 %)	4 (21 %)	
	Grade 3	10 (12 %)	11 (12 %)	6 (32 %)	
	Grade 4	51 (60 %)	64 (69 %)	7 (37 %)	
Endovascular aneurysm treatment, n (%)		53 (62 %)	68 (73 %)	7 (37 %)	0.12
Delayed cerebral ischemia, n (%)		38 (45 %)	50 (54 %)	7 (37 %)	0.22
Glasgow Outcome Scale at 12 months*, n (%)					0.15
	Dead	25 (30 %)	10 (11 %)	0 (0 %)	
	Neurodegenerative state	0 (0 %)	1 (1 %)	0 (0 %)	
	Severe disability	14 (17 %)	26 (30 %)	4 (21 %)	
	Moderate disability	22 (27 %)	25 (28 %)	5 (26 %)	
	Good recovery	22 (27 %)	26 (30 %)	10 (53 %)	

* Missing for 7 patients
